# Hepatic-associated vascular morphological assessment to predict overt hepatic encephalopathy before TIPS: a multicenter study

**DOI:** 10.1007/s12072-024-10686-2

**Published:** 2024-06-04

**Authors:** Xiaoqiong Chen, Mingsheng Huang, Xiangrong Yu, Jinqiang Chen, Chunchun Xu, Yunzheng Jiang, Yiting Li, Yujie Zhao, Chongyang Duan, Yixin Luo, Jiawei Zhang, Weifu Lv, Qiyang Li, Junyang Luo, Dandan Dong, Taixue An, Ligong Lu, Sirui Fu

**Affiliations:** 1 Zhuhai Interventional Medical Centre, Zhuhai Clinical Medical College of Jinan University (Zhuhai People’s Hospital), No. 79 Kangning Road, Zhuhai, 519000 Guangdong Province China; 2 Zhuhai Engineering Technology Research Center of Intelligent Medical Imaging, Zhuhai Clinical Medical College of Jinan University (Zhuhai People’s Hospital), No. 79 Kangning Road, Zhuhai, 519000 Guangdong Province China; 3 Department of Interventional Radiology, The Third Affiliated Hospital of Sun Yat-Sen University, Guangzhou, China; 4 Department of Radiology, Zhuhai Clinical Medical College of Jinan University (Zhuhai People’s Hospital), Zhuhai, China; 5 Department of Biostatistics, School of Public Health, Southern Medical University, Guangzhou, China; 6 Interventional Radiology Department, Division of Life Sciences and Medicine, The First Affiliated Hospital of USTC, University of Science and Technology of China, Hefei, China; 7 Department of Interventional Radiology, Shenzhen People’s Hospital, Shenzhen, China; 8 Department of Laboratory Medicine, Nanfang Hospital, Southern Medical University, No. 1023-1063 Shatai Road, Guangzhou, 510515 Guangdong Province China; 9 Guangdong Provincial Key Laboratory of Tumor Interventional Diagnosis and Treatment, Zhuhai Institute of Translational Medicine, Zhuhai, China

**Keywords:** Transjugular intrahepatic portosystemic shunt, Overt HE, Prediction, Vascular, Morphological change, Discrimination, Calibration, Subgroups, Populations, Applet

## Abstract

**Background:**

To provide patients the chance of accepting curative transjugular intrahepatic portosystemic shunt (TIPS) rather than palliative treatments for portal hypertension-related variceal bleeding and ascites, we aimed to assess hepatic-associated vascular morphological change to improve the predictive accuracy of overt hepatic encephalopathy (HE) risks.

**Methods:**

In this multicenter study, 621 patients undergoing TIPS were subdivided into training (413 cases from 3 hospitals) and external validation datasets (208 cases from another 3 hospitals). In addition to traditional clinical factors, we assessed hepatic-associated vascular morphological changes using maximum diameter (including absolute and ratio values). Three predictive models (clinical, hepatic-associated vascular, and combined) were constructed using logistic regression. Their discrimination and calibration were compared to test the necessity of hepatic-associated vascular assessment and identify the optimal model. Furthermore, to verify the improved performance of Model^C−V^, we compared it with four previous models, both in discrimination and calibration.

**Results:**

The combined model outperformed the clinical and hepatic-associated vascular models (training: 0.814, 0.754, 0.727; validation: 0.781, 0.679, 0.776; *p* < 0.050) and had the best calibration. Compared to previous models, Model^C−V^ showed superior performance in discrimination. The high-, middle-, and low-risk populations displayed significantly different overt HE incidence (*p* < 0.001). Despite the limited ability of pre-TIPS ammonia to predict overt HE risks, the combined model displayed a satisfactory ability to predict overt HE risks, both in the low- and high-ammonia subgroups.

**Conclusion:**

Hepatic-associated vascular assessment improved the predictive accuracy of overt HE, ensuring curative chances by TIPS for suitable patients and providing insights for cirrhosis-related studies.

**Graphical Abstract:**

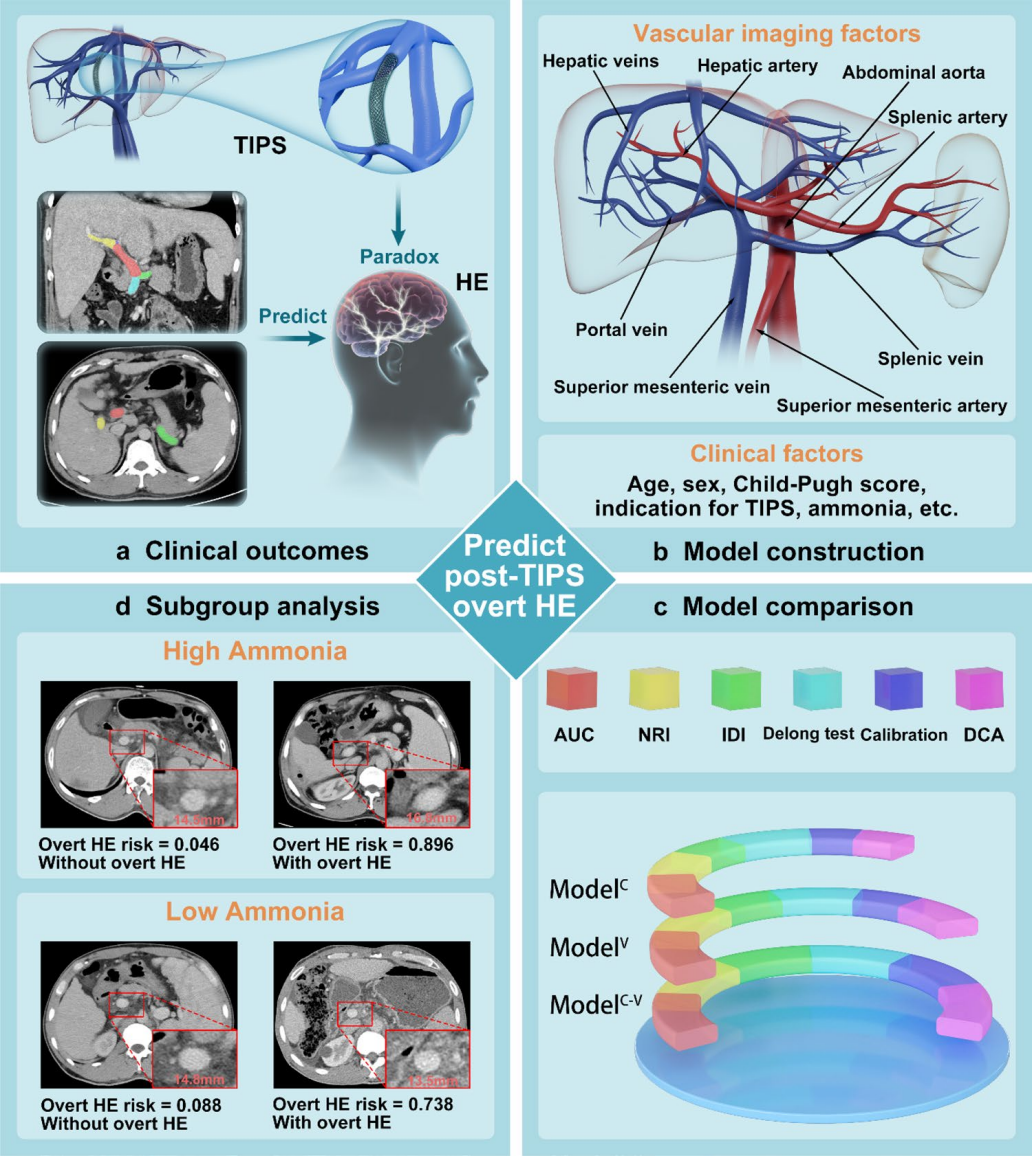

**Supplementary Information:**

The online version contains supplementary material available at 10.1007/s12072-024-10686-2.

## Introduction

Portal hypertension can lead to major clinical complications, including variceal bleeding and ascites [1]. Transjugular intrahepatic portosystemic shunt (TIPS) establishes artificial channels between the hepatic and portal veins to reduce the pressure in the portal vein [2, 3]. Notably, rather than merely offering palliative effects, TIPS is the only minimally invasive method that can decrease and even normalize portal pressure, thereby providing a curative effect in controlling variceal bleeding and refractory ascites [3, 4]. However, the American, Chinese, and European guidelines all recommend endoscopic therapy, non-selective β-blockers, and paracentesis as first-line therapies for portal hypertension-related variceal bleeding and ascites, while only recommending TIPS as an alternative option [1, 5–8]. One major reason for this is that TIPS can cause overt hepatic encephalopathy (HE), with an incidence ranging from 10 to 50% [9–11], that can negatively impact the quality of life and increase the mortality of patients [2, 12]. Moreover, even after multiple treatments, the recurrence rate of HE is still high. All these considerations make predicting overt HE risks an essential step to ensure that suitable patients have a chance for curative TIPS.

Predicting HE has always been a focal point in cirrhosis-related studies [13, 14]. Previous studies focused on clinical or biological factors, which were mainly related to liver function [15, 16]. However, the normality of the hepatic-associated vascular system (portal, arterial, and venous) is the cornerstone of liver function. An increase in the average portal vein diameter may be considered an indicator of portal hypertension. When portal hypertension occurs, increased flow, increased vascular resistance, or a combination of both may influence portal diameter and affect the detoxification of toxic substances (especially plasma ammonia), thus influencing the risk of overt HE [17, 18]. In this regard, our previous study [19] demonstrated that morphological assessments of the liver and spleen were feasible and could be used for predicting post-TIPS overt HE. Therefore, morphological assessment of the hepatic-associated vascular system may provide additional information for predicting post-TIPS overt HE. In addition, the hepatic-associated vascular morphological assessment may have an impact on the clinical use of the stent size to control hepatic portal-venous perfusion and clinical decision-making considering the management of TIPS stents.

Considering the necessity and feasibility of morphological assessment of the hepatic-associated vascular system, this study aimed to use the inflow and outflow vasculature related to the liver to increase the predictive accuracy of post-TIPS overt HE.

## Materials and methods

### Patient selection

**Fig. 1:**
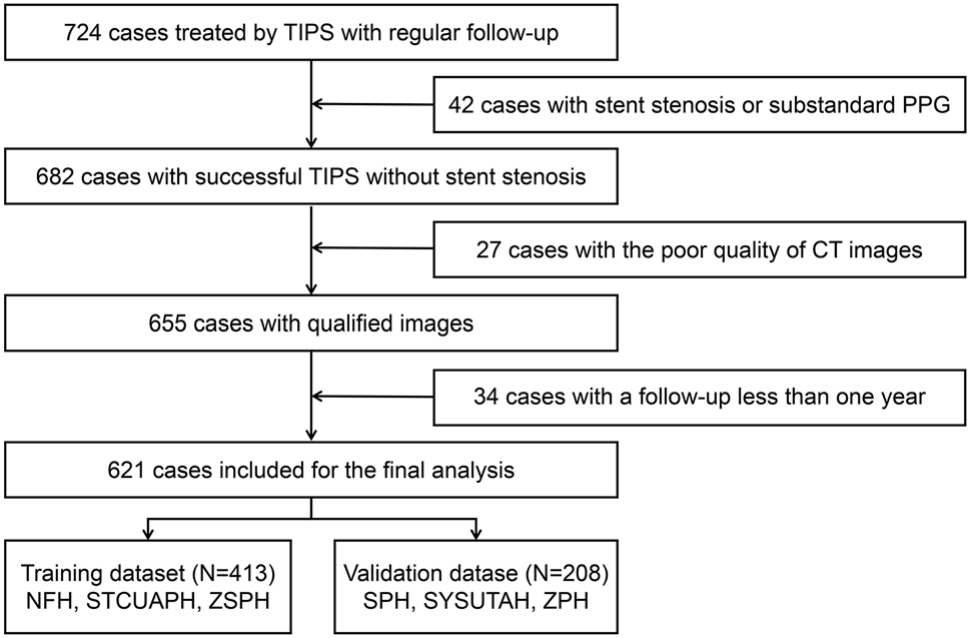
Inclusion and exclusion flowcharts. The inclusion and exclusion flowcharts show patient selection for this study. We screened 724 patients from 6 hospitals. After inclusion and exclusion criteria were applied, 621 patients were included and divided into the training dataset (413 cases) and external validation dataset (208 cases). *TIPS* transjugular intrahepatic portosystemic shunt, *PPG* portal pressure gradient, *CT* computed tomography, *NFH* Nanfang Hospital, *STCUAPH* the First Affiliated Hospital of the University of Science and Technology of China, *ZSPH* Zhongshan City People’s Hospital, *SPH* Shenzhen People’s Hospital, *SYSUTAH* the Third Affiliated Hospital of Sun Yat-sen University, *ZPH* Zhuhai People’s Hospital

This retrospective multicenter study including 621 patients between January 2012 and January 2022 was conducted at 6 hospitals in China: Nanfang Hospital (NFH), The First Affiliated Hospital of the University of Science and Technology of China (STCUAPH), Zhongshan City People’s Hospital (ZSPH), Shenzhen People’s Hospital (SPH), The Third Affiliated Hospital of Sun Yat-sen University (SYSUTAH), and Zhuhai People’s Hospital (ZPH). All patients underwent TIPS treatment because of variceal rebleeding and/or refractory ascites. The inclusion criteria were as follows: (1) at least one variceal rebleeding or refractory ascites after therapies, such as endoscopic treatment, vasoactive drugs, or large-volume paracentesis; (2) regular follow-up for at least 1 year; (3) age ≥ 18 years; (4) a Child–Pugh score ≤ 13 points and no liver failure; and (5) a portosystemic pressure gradient decrease > 50% from baseline or < 12 mmHg after TIPS [6, 8, 9]. The exclusion criteria were as follows: (1) TIPS performed to prevent failure or rebleeding after initial pharmacological and endoscopic therapy (early TIPS); (2) stent stenosis or occlusion during follow-up; (3) hepatocellular carcinoma that is not consistent with the Milan criteria for transplantation, that is, a single lesion < 3 cm or fewer than three lesions with the largest measuring ≤ 3 cm (per the Milan criteria [7], a patient with hepatocellular carcinoma is eligible for liver transplantation if they have either a single lesion ≤ 5 cm or two to three lesions, each ≤ 3 cm); and (4) patients with previous overt HE. Based on the inclusion and exclusion criteria, 621 participants were enrolled in this study (Fig. 1).

The study was approved by The Ethics Review Committee of the Zhuhai People’s Hospital. The requirement for informed consent was waived because the patients’ data were collected retrospectively. All patient data were anonymized before analysis.

### Preoperative treatment and TIPS procedure

Based on the guidelines, the following preoperative treatments were performed if necessary: (1) anemia and coagulopathy were corrected to ensure patient safety during TIPS treatment (hemoglobin > 7 g/dL and prothrombin time < 25 s); (2) abdominal paracentesis was performed before TIPS to prevent massive hemorrhage; (3) vasoactive drugs (terlipressin [2 mg/4 h], somatostatin [250–500 μg/h], or octreotide [25–50 μg/h]) and prophylactic antibiotics (ceftriaxone [1 g/24 h]) were administered before TIPS [1].

According to the practice guidelines for TIPS and a previously described protocol, all procedures were performed by experienced physicians in interventional radiology. All TIPS treatments were performed using a standard technique guided by digital subtraction angiography. Polytetrafluoroethylene-covered stents measuring 8 mm were used in all patients, and an 8-mm balloon dilatation was performed to expand the stent to 8 mm (thereby preventing the possibility of passive expansion of stents after TIPS). Portosystemic pressure gradient reductions of > 50% from baseline or < 12 mmHg were considered successful [2].

### Follow-up and outcomes

Following the guidelines, oral medicines such as lactulose or rifaximin were not administered to patients after TIPS until HE occurred [9]. For the included patients, the baseline demographic characteristics and computed tomography (CT) images were collected within 7 days before TIPS. Follow-up was performed once a week for the first month, followed by telephone interviews, outpatient visits, or hospital visits every 4 weeks. Patients and their families were asked to contact the physician immediately in case of alterations in the patient’s mental state. The symptoms of HE, such as lethargy, apathy, and obvious changes in personality, were recorded in detail. After repeated confirmations, the stage and degree of HE were evaluated.

The study outcome was post-TIPS overt HE, which was defined as grades II, III, and IV according to the West Haven Criteria (Table S1) [9]. For patients without overt HE, follow-up was continued every 4 weeks until liver transplantation, death, or the end of the study (March 2023).

### Image acquisition and hepatic-associated vascular assessment

Based on our previous work [19–21], CT images were found to be more suitable for multicenter studies. Thus, in this study, we performed hepatic-associated vascular assessment using CT images. The CT scanning parameters and contrast agent injection in each collaborative hospital are listed in Table S2. The image data were transferred directly to the picture archiving and communication system.

Data for analysis were measured on arterial and portal-venous phase images because the vessel could be more clearly visualized for subsequent measurements. The diameters of the portal-venous and arterial systems were measured independently by two radiologists (Q.L and X.Y with 10 and 20 years of experience in medical radiology, respectively). All measurements were performed blindly (Text S1). The intra- and inter-observer agreements in the hepatic vascular diameters are listed in Table S3. The intra- and inter-observer agreements of the measurements were excellent; the intra-observer correlation coefficient *R* values ranged from 0.929 to 0.980 and inter-observer correlation coefficient R values ranged from 0.944 to 0.999.

### Clinical and hepatic-associated vascular factors

Clinical factors, such as age, sex, and Child–Pugh score, as well as indications for TIPS, of candidates are listed in Table S4.

Two parameter categories were used to assess hepatic-associated vascular morphological changes. The first included absolute value indicators such as the maximum diameters of the portal vein and splenic artery (Table S5). The second included ratio value indicators, such as the diameter ratio of the portal and splenic veins to control potential bias introduced by physiological differences (Table S5).

## Statistical analysis

Patients from three centers were considered as the training dataset, while the remaining patients from the three other centers were used as the external validation dataset. For the comparison of those two datasets, quantitative data expressed as means (standard deviations) or medians (interquartile ranges) based on their distribution were compared using the *t* test or Wilcoxon rank sum test appropriately, and categorical variables presented as percentages were compared using Pearson’s Chi-squared test or Fisher’s exact test as appropriate.

For model construction, using the training dataset, the Lasso was applied to screen the clinical and hepatic-associated vascular morphological factors related to the risks of post-TIPS overt HE. Subsequently, three models were developed: the clinical model (Model^C^) using the identified clinical factors, the hepatic-associated vascular model (Model^V^) using the identified hepatic-associated vascular morphological change factors, and the combined model (Model^C−V^) using both the identified clinical and hepatic-associated vascular morphological factors.

In both the training and validation datasets, the discrimination of those three models was compared using net reclassification improvement (NRI), integrated discrimination improvement (IDI), and the receiver operating characteristic (ROC) analysis followed by the Delong test. The calibration was compared using the calibration plot. We also performed a decision curve analysis (DCA) to compare their clinical utility. An applet was then constructed for the best model. Subgroup analysis based on the pre-TIPS sex, Child–Pugh score, or indication for TIPS was also performed to further test the stability of our model in different subgroups. Specifically, we analyzed the risk of patients with high and low ammonia levels of suffering from overt HE.

All statistical analyses were performed in R (version 4.2.1). A two-sided *p* < 0.050 was considered statistically significant. Our study strictly followed the Transparent Reporting of a multivariable prediction model for Individual Prognosis or Diagnosis statement.

## Results

### Patient characteristics

Overall, 621 patients were included in our study and divided into the training (413 patients from 3 hospitals [NFH, STCUAPH, ZSPH]) and external validation datasets (208 patients from 3 other hospitals including [SPH, SYSUTAH, ZPH]) (Table 1). The symptoms leading to TIPS included variceal bleeding (510 patients; training, 340 patients; validation, 170 patients) and refractory ascites (111 patients; training, 73 patients; validation, 38 patients). Overt HE occurred in 188 patients (training: 131 patients, validation, 57 patients) (Table S6). Baseline characteristics of the patients are reported in Table 1. A total of 621 patients were included in the survival rate analysis (training group: *N* = 413; validation group: *N* = 208). Forty-three patients died after TIPS (training, *N* = 21; validation, *N* = 22). The 1 year survival rate was 93.0% (training: 95.0%; validation: 89.5%), consistent with that in a previous study [22].

**Table 1:** Baseline demographics of patients

Clinical factors	Training dataset (*N* = 413)	Validation dataset (*N* = 208)	*p* value
Age (year)	53.0 ± 11.1	52.8 ± 12.0	0.822
Sex (N)			0.575
Male	320 (78%)	166(80%)	
Female	93 (22%)	42(20%)	
Etiology (N)			< 0.001*
Alcohol	168 (41%)	40 (19%)	
Hepatitis B/C	171 (41%)	131 (64%)	
Cholestatic	11 (3%)	3 (1%)	
Others	63 (15%)	34 (16%)	
Child–Pugh score (point)	8 (6, 9)	8 (7, 9)	0.106
MELD score (point)	10 (8,13)	10 (9,12)	0.906
ALT	20.0 (14.0, 33.0)	20.0 (14.0, 29.0)	0.423
AST	29.0 (21.0, 41.0)	28.0 (22.0, 40.0)	0.697
Direct bilirubin (mg/dL)	0.5 (0.4, 0.9)	0.5 (0.3, 0.8)	0.004*
Indirect bilirubin (mg/dL)	0.5 (0.4, 0.8)	0.5 (0.4, 0. 8)	0.551
Albumin (g/L)	33.3 ± 5.3	34.1 ± 4.9	0.584
Serum sodium (mmol/L)	140.0 (137.0, 142.0)	140.0 (138.0, 142.0)	0.115
INR	1.2 (1. 1, 1.4)	1.3 (1.2, 1.5)	0.001*
Ammonia (μmol/L)			< 0.001*
< 72.0	337 (82%)	199 (96%)	
≥ 72.0	76 (18%)	9 (4%)	
Indication for TIPS(N)			0.943
Variceal bleeding	340 (82%)	170 (82%)	
Refractory ascites	73 (18%)	38 (18%)	
Liver cancer			0.058
Yes	49 (12%)	37 (18%)	
No	364 (88%)	171 (82%)	
Diabetes			0.635
Yes	85 (21%)	47 (23%)	
No	328 (79%)	161 (77%)	

Normally distributed factors are expressed using means ± standard deviations; non-normally distributed factors are expressed as medians (interquartile ranges)

*MELD* model for end-stage liver disease, *ALT* alanine aminotransferase, *AST* aspartate aminotransferase, *INR* international normalized ratio, *TIPS* transjugular intrahepatic portosystemic shunt

^*^With a *p* < 0.050

### Development of the clinical, vascular, and combined models

Overall, 45 preliminary factors, including 20 clinical factors (such as age, sex, and Child–Pugh score) and 25 hepatic-associated vascular morphological factors (14 absolute value factors, and 11 ratio value factors) were screened (Tables S4 and S5). After the univariate (Tables S7 and S8) and multivariate logistic regression (Table S9 and S10), the clinical model (Model^C^), including age (odds ratio: 1.014; 95% CI 0.993, 1.036; *p* = 0.182) and Child–Pugh score (odds ratio: 1.803; 95% CI 1.547, 2.100; *p* < 0.001); the hepatic-associated vascular model (Model^V^), including the maximum diameter of the portal vein (odds ratio: 1.097; 95% CI 1.011, 1.191; *p* = 0.027), diameter ratio of the portal and splenic veins (odds ratio: 5.205; 95% CI 2.878, 9.415; *p* < 0.001), and diameter ratio of the portal and middle hepatic veins (odds ratio: 0.144; 95% CI 0.030, 0.699; *p* = 0.016); and the combined model (Model^C−V^), including age (odds ratio: 1.015; 95% CI 0.993, 1.038; *p* = 0.185), Child–Pugh score (odds ratio: 1.825; 95% CI 1.548, 2.152; *p* < 0.001), the maximum diameter of the portal vein (odds ratio: 1.200; 95% CI 1.099, 1.310; *p* < 0.001), and diameter ratio of the portal and splenic veins (odds ratio: 3.500; 95% CI 1.891, 6.481; *p* < 0.001) were identified by multivariate regression (Table S9).

### Necessary test of combined clinical and vascular factors

**Fig. 2:**
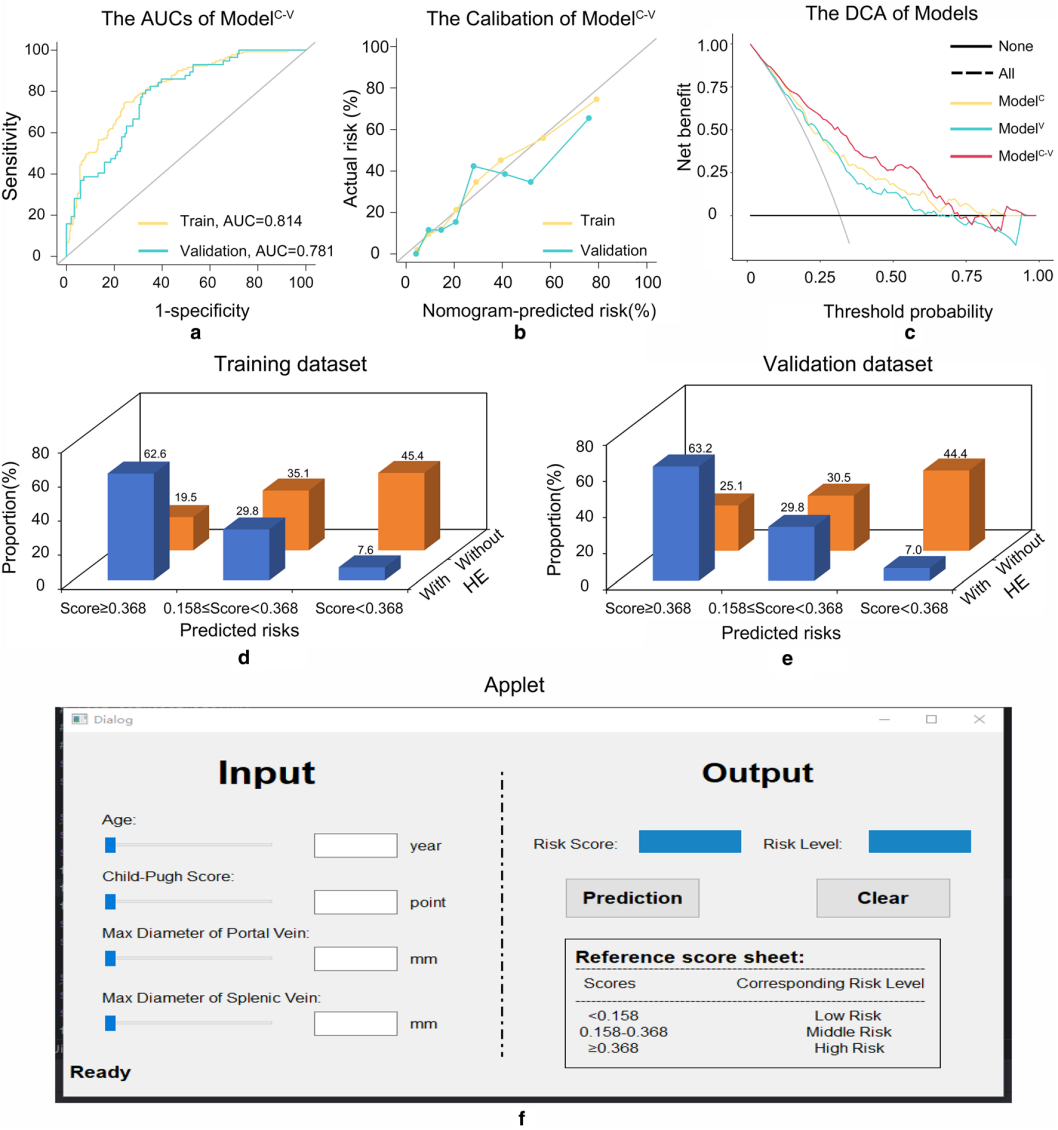
Performance of the combined model and its applet. Evaluation of the performance of the combined model. **a** Receiver operating characteristic (ROC) curve analysis showing the area under the curve (AUC) for the combined model in the training (AUC: 0.814) and validation (AUC: 0.781) datasets. **b** Calibration plot demonstrating the performance of the combined model. **c** Decision curve analysis (DCA) comparing the combined model with three other models. When divided by the cut-off value of Model^C−V^ (score of 0.158 and 0.368), the high-, middle, and low-risk populations had statistically significant differences (both *p* < 0.001) in the training (**d**) and validation (**e**) datasets. Examples of patients classified as different risks using the combined model at the applet (**f**). *HE* hepatic encephalopathy

For discrimination, Model^C−V^ had better areas under the curves (AUCs) than Model^C^ and Model^V^ (training dataset: 0.814 vs 0.754 and 0.727; validation dataset: 0.781 vs. 0.679 and 0.776), with statistical differences based on the Delong test, NRI, and/or IDI (Fig. S1 and Table S11). For calibration, Model^C−V^ was comparable to Model^V^ but superior to Model^C^ (Fig. S1). Based on these results, Model^C−V^ was chosen as the final model (Fig. 2a, b). Regarding the DCA, Model^C−V^ performed better than Model^C^ and Model^V^ (Fig. 2c).

### Model performance and comparison

We evaluated the performance of Model^C−V^ in discrimination compared to those of previous models [23, 24], according to the Delong test, NRI, and IDI. Transversal psoas muscle thickness (TPMT) and psoas muscle attenuation (PMA) were measured at the level of the third lumbar vertebrae (L3). After excluding 174 patients whose full L3 transverse process could not be obtained, 447 patients were included for the analysis of combined TPMT and PMA (TPMT and PMA model) (training dataset: 339 patients, validation dataset: 108 patients).

Model^C−V^ demonstrated significant improvement over the previous models in the training dataset (Table 2, Fig. S2-a): albumin (ALB) (AUC = 0.566, *p* < 0.001); Child–Pugh (AUC = 0. 746, *p* < .001); model for end-stage liver disease (MELD) (AUC = 0.578, *p* < 0.001); TPMT and PMA (AUC = 0. 599, *p* < 0.001). Similar results were observed for the validation dataset (Table 2, Fig. S2-b): ALB (AUC = 0. 605, *p* < 0.001); Child–Pugh (AUC = 0.647, *p* < 0.001); MELD (AUC = 0. 619, *p* < 0.001); TPMT and PMA (AUC = 0. 650, *p* < 0.001).

**Table 2:** Pairwise comparison of the models

	AUC	Delong test	NRI	IDI
Training dataset				
ALB vs. Model^C−V^	0.566 vs.0.814	< 0.001^*^	< 0.001^*^	< 0.001^*^
Child–Pugh vs. Model^C−V^	0.746 vs.0.814	< 0.001^*^	< 0.001^*^	< 0.001^*^
MELD vs. Model^C−V^	0.578 vs.0.814	< 0.001^*^	< 0.001^*^	< 0.001^*^
TPMT and PMA vs. Model^C−V^	0.599 vs.0.814	< 0.001^*^	< 0.001^*^	< 0.001^*^
Validation dataset				
ALB vs. Model^C−V^	0.605 vs.0.814	< 0.001^*^	0.004^*^	< 0.001^*^
Child–Pugh vs. Model^C−V^	0.647 vs.0.814	< 0.001^*^	0.002^*^	< 0.001^*^
MELD vs. Model^C−V^	0.619 vs.0.814	< 0.001^*^	< 0.001^*^	< 0.001^*^
PMT and PMA vs. Model^C−V^	0.650 vs.0.814	< 0.049^*^	0.022^*^	< 0.001^*^

Data are presented as *p* values

*NRI* net reclassification improvement, *IDI* integrated discrimination improvement, *ALB* albumin, *MELD* model for end-stage liver disease, *PMT* transversal psoas muscle thickness, *PMA* psoas muscle attenuation, *TPMT* transversal psoas muscle thickness, *AUC* area under the curve

^*^(*p* < 0.050 is significant)

### Stability tests and risk stratification

**Fig. 3:**
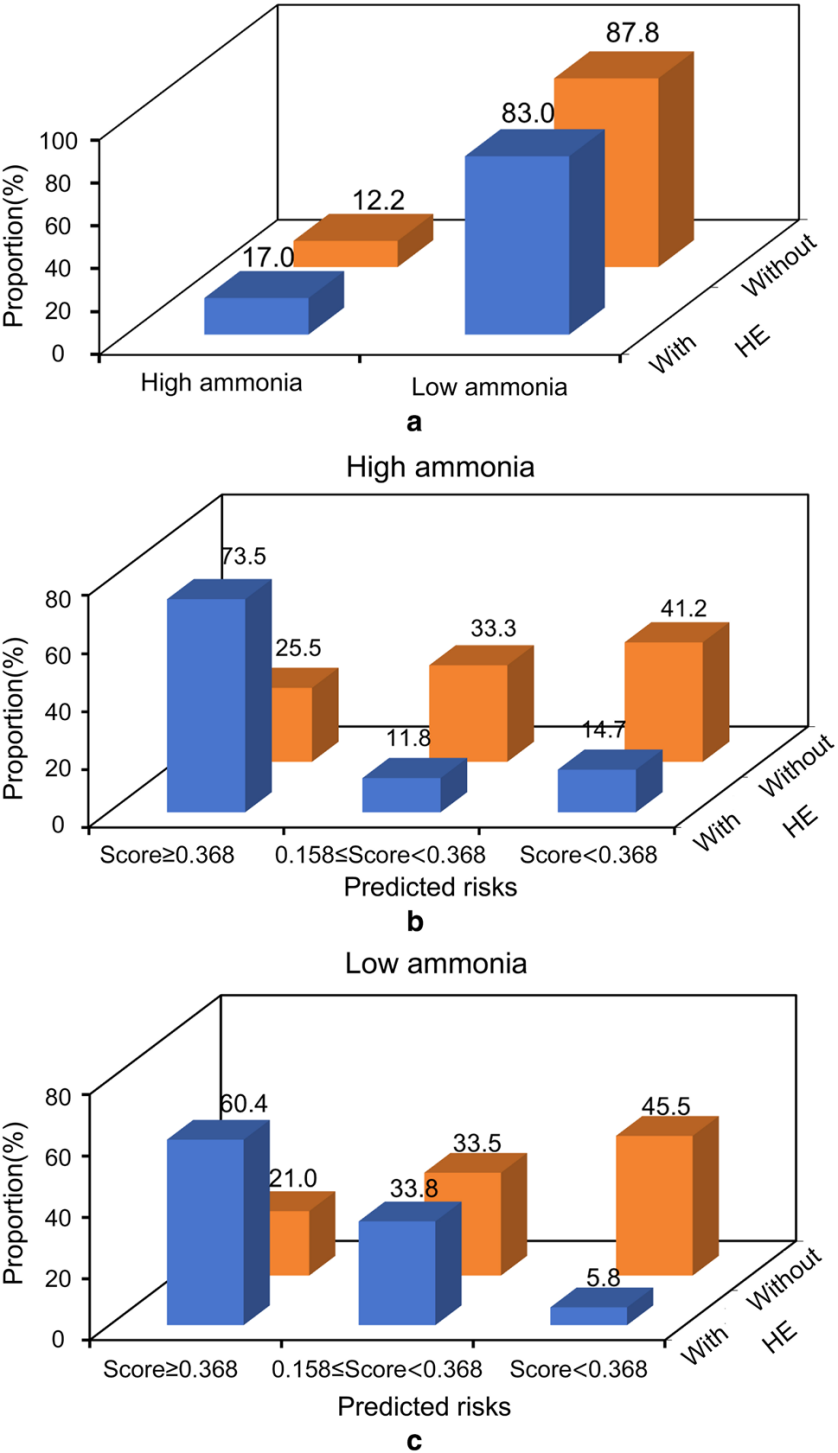
Model^C−V^ rather than pre-TIPS ammonia could stratify patients for their risk of overt HE. Subdivided by the level of pre-TIPS ammonia, the subgroups had limited differences regarding the risk of overt HE (**a**). However, subdivided by our Model^C−V^, the subgroups had significant differences in the risk of overt HE, both in the high ammonia (**b**) and low ammonia populations (**c**). *TIPS* transjugular intrahepatic portosystemic shunt, *HE* hepatic encephalopathy

To assess comparability among different subgroups, we investigated whether preoperative ammonia, sex, Child–Pugh score, and the indication for TIPS could influence the performance of Model^C−V^. The results showed no statistical difference among the populations (Table S12): preoperative ammonia < 72.0 vs. ≥ 72.0 (0.804 vs. 0.810; *p* = 0.913; Fig. S3-a); male vs. female (0.796 vs. 0.833; *p* = 0.372; Fig. S3-b); Child–Pugh score < 8 vs. ≥ 8 (0.809 vs. 0.748; *p* = 0.138; Fig. S3-c); and variceal bleeding vs. refractory ascites (0.812 vs. 0.750; *p* = 0.223; Fig. S3-d). The discriminating ability to predict overt HE was similar in patients with different ammonia levels. The different pre-TIPS ammonia levels had limited ability to predict post-TIPS overt HE. However, the combined model could stratify pre-TIPS ammonia for their post-TIPS overt HE risks (Fig. 3).

**Fig. 4:**
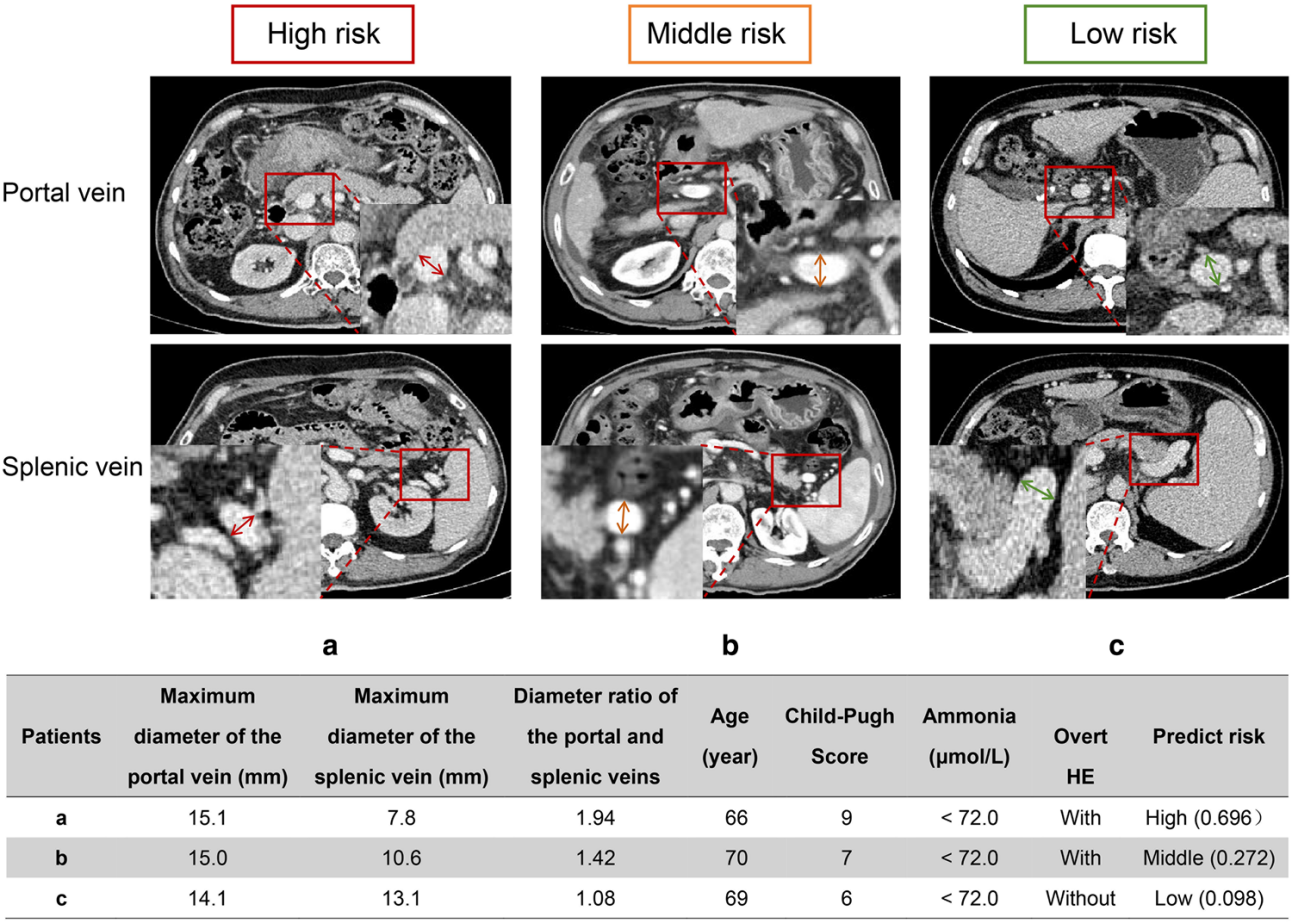
Images of hepatic-associated vascular morphological changes in patients with high, middle, and low risks. Vascular imaging characteristics of a high-risk patient: maximum diameters of the portal vein and splenic vein (**a**). Vascular imaging characteristics of a middle-risk patient: maximum diameters of the portal vein and splenic vein (**b**). Vascular imaging characteristics of a low-risk patient: maximum diameters of the portal vein and splenic vein (**c**). The table shows the characteristics of three patients. *HE* hepatic encephalopathy

Based on the AUC of Model^C−V^, by trisect score sorted from smallest to largest, the cut-off values of 0.368 and 0.158 were identified. The proportion of overt HE was significantly different between the high-risk (Model^C−V^ score ≥ 0.368), middle-risk (0.158 ≤ Model^C−V^ score < 0.368), and low-risk (Model^C−V^ score < 0.158) populations in the training (62.6% vs. 29.8% vs. 7.6%; *p* < 0.001; Fig. 2d) and validation (63.2% vs. 29.8% vs. 7.0%; *p* < 0.001; Fig. 2e) datasets. The demographics of high-, middle-, and low-risks population are shown in Table S13 and Table S14. The number of patients who died after being enrolled in our model had a limited impact on the incidence of post-TIPS overt HE, as shown in Fig. S4. Accordingly, an applet for Model^C−V^ was constructed (Fig. 2f; https://github.com/FuSirui123/predict-overt-hepatic-encephalopathy). Examples of populations with different risks are shown in Fig. 4.

## Discussion

In our study, the combined model based on the hepatic-associated vascular morphological assessment significantly improved the performance of the model in predicting overt HE. In particular, rather than pre-TIPS ammonia, the combined model could truly stratify patients for their overt HE risks. Combining these findings, our model could be used to assist patient selection for TIPS and provide additional insight for studies related to TIPS and even cirrhosis.

For variceal bleeding and ascites caused by portal hypertension, two treatments are available, including first-line treatments, such as large-volume paracentesis [25] and non-selective β-blockers [26], and alternative options, such as TIPS [5]. TIPS has not been recommended as the first-line therapy because it may cause an increase in some toxic substances in the central nervous system, leading to post-TIPS HE, particularly overt HE, which significantly negatively affects the quality of life and increases mortality of patients [1, 6–8, 27]. A prior study demonstrated that post-TIPS overt HE could be predicted via changes in the liver, allowing more patients to accept TIPS [19].

Hepatic encephalopathy is closely associated with blood, especially hepatic-associated vasculature; hence, we assessed the hepatic-associated vascular morphological changes. The splenic component represents only approximately 40% of portal blood flow in healthy patients and exceeds 50% in patients with cirrhosis. This confirms the determinant role of splenic blood flow in the context of enhanced portal blood flow, which contributes to portal hypertension [28]. Furthermore, factors influencing blood flow in the portal system can cause opposite variations in hepatic artery blood flow (“buffer response”), which tends to attenuate alterations in total hepatic blood flow [17]. Changes in the hepatic vascular system are essential pathophysiological characteristics of portal hypertension. Therefore, non-invasive imaging-based assessment of hepatic vascular parameters has the potential to yield meaningful imaging markers for clinically significant portal hypertension identification. Portal diameter may identify more severe portal hypertension causing more blood flow bypassing the liver after TIPS and, therefore, more HE. Therefore, hepatic-associated vascular morphological changes that quantitatively predict the risk of overt HE can provide crucial information that can be used to guide the decision to perform TIPS.

To address this, we combined traditional clinical and hepatic-associated vascular morphological factors designed to assess the severity of cirrhosis and portal hypertension. Regarding clinical factors, age and Child–Pugh score were related to post-TIPS overt HE, which is consistent with the findings of recent studies [5, 29].

In the hepatic-associated vascular morphological change of patients with and without overt HE (Table S15), many factors were correlated with overt HE. The results of Lasso to screen the factors showed that the maximum diameters of the portal vein, as well as the diameter ratio of the portal and splenic veins, were most related to post-TIPS overt HE. We conducted a thorough analysis to assess this correlation and found that the type of collaterals and maximum diameter of the splenic vein did not demonstrate significance in univariate logistic regression (Tables S8 and S16). This may be because the assessment of the vasculature is not sufficiently precise.

Regarding hepatic-associated vascular morphological factors, the identified parameters included two categories: the maximum diameter of the portal vein and the diameter ratio of the portal and splenic veins. The diameter ratio of the portal and splenic veins could control the impact of individual differences on the outcomes, which formed a supplement. The increased diameter of the portal vein and the increased diameter ratio of the portal and splenic veins showed that more blood from the superior mesenteric vein flowed into the portal vein before TIPS. As toxic substances (especially plasma ammonia) in the intestinal system were mainly absorbed in the superior mesenteric vein after TIPS, more undetoxified portal vein blood flows directly into the nervous system through the shunt vessel, thereby bringing more ammonia into the brain [30]. The difference in stent diameter may influence the flow from the portal vein to the hepatic vein, thus affecting the detoxification of toxic substances in the liver, and more undetoxified portal vein blood flows directly into the nervous system through the shunt vessel, increasing the risk of overt HE. Our study focused on the assessment of hepatic-associated vascular changes, and the portal diameter may influence the clinical use of the stent size to control hepatic portal-venous perfusion and clinical decision-making considering the management of TIPS stents.

The performance of the clinical model needed to be further improved (Fig. S1). Thus, we combined the clinical and hepatic-associated vascular morphological factors. The discrimination and calibration of the combined Model^C−V^ were significantly better than those of Model^C^ and Model^V^. Furthermore, the AUCs of Model^C−V^ were significantly higher than those of Model^V^ in the training dataset (0.814 vs. 0.727), while the AUCs of Model^V^ were similar to those of Model^V^ in the validation dataset (0.781 vs. 0.776). The performances of NRI, IDI, and the Delong test were statistically superior; and there were improvements in the calibration and DCA results. These results revealed the importance of including hepatic-associated vascular morphological factors in predicting post-TIPS overt HE. We observed that Model^C−V^ outperformed Model^V^ in NRI, IDI, and DCA. These results demonstrated that hepatic-associated vascular morphological change contributed to improvements in the model. Based on these results, clinical and hepatic-associated vascular morphological factors are indispensable for predicting post-TIPS overt HE.

This study has some limitations. First, the vasculature was manually segmented in 2D slices because we did not solve the imbalance problems that may affect the accuracy of the automatic 3D segmentation: foreground (vascular system) vs. background (other regions such as the liver parenchyma and stomach) and thin vs. thick vessels. A more accurate 3D assessment of the vascular system, such as that of the splenic vein is needed. With continued efforts, we will be able to perform accurate automatic 3D segmentation of vascular systems in the future. Second, the retrospective design and focus on Eastern patients limits our study. Our study can neither assess occult HE nor be applied to Western patients, which should also be studied in future prospective studies. Third, patients without an 8-mm polytetrafluoroethylene-covered stent were excluded to control for possible confounding factors. Therefore, whether our conclusion applies to patients with a 10 mm polytetrafluoroethylene-covered stent or bare metal stent requires further investigation [9, 31]. Fourth, we only analyzed the quality of the major portosystemic shunts and did not analyze the quantification, such as the diameter or area, which needs to be further explored in our study. Fifth, as our patients were included before the publication of the 2022 EASL guidelines, the change from 2014 to 2022 EASL guidelines [9, 32], such as the use of prophylaxis, should be further explored to test whether it would have an influence on model performance.

In conclusion, we constructed a combined model that could predict post-TIPS overt HE based on the hepatic-associated vascular morphological assessment. Assisted by our model, patients with low risk may be able to accept alternative TIPS treatment.

### Supplementary Information

Below is the link to the electronic supplementary material.

Supplementary file1 (DOCX 1319 KB)

## Data Availability

Owing to privacy concerns, the data related to patients are not available for public access but can be obtained from the corresponding author on reasonable request, subject to approval by the institutional review board of Zhuhai People’s Hospital.

## Abbreviations

TIPS: Transjugular intrahepatic portosystemic shunt
HE: Hepatic encephalopathy
CT: Computed tomography
PPG: Portal pressure gradient
NRI: Net reclassification improvement
IDI: Integrated discrimination improvement
ROC: Receiver operating characteristic
DCA: Decision curve analysis
AUC: Area under the curve
ALB: Albumin
MELD: Model for end-stage liver disease
TPMT: Transversal psoas muscle thickness
PMA: Psoas muscle attenuation
