# Supplementary material for: Hepatic-associated vascular morphological assessment to predict overt hepatic encephalopathy before TIPS: a multicenter study
Source: Hepatol Int. 2024 Jun 4;18(4):1238–48. doi: 10.1007/s12072-024-10686-2 (PMC11297904; doi:10.1007/s12072-024-10686-2)
Supplement: Supplementary file 1 — Supplementary file1 (DOCX 1319 KB) [file 12072_2024_10686_MOESM1_ESM.docx]

**Hepatic-associated vascular morphological assessment to predict overt hepatic encephalopathy before TIPS: a multicenter study**

Xiaoqiong Chen *; Mingsheng Huang *; Xiangrong Yu *; Jinqiang Chen *; Chunchun Xu; Yunzheng Jiang; Yiting Li; Yujie Zhao; Chongyang Duan; Yixin Luo; Jiawei Zhang; Weifu Lv; Qiyang Li; Junyang Luo; Dandan Dong; Taixue An ^#^; Ligong Lu ^#^; Sirui Fu ^#^.

**Supplementary material**

**Table of contents**

1. Table S1. Clinical characterization of overt HE
2. Table S2. Major parameters of CT image acquisition
3. Text S1: Vascular measured methodology
4. Table S3. Evaluation of the intra- and interobserver agreements
5. Table S4. Candidate clinical factors
6. Table S5. Candidate vascular factors
7. Table S6. Baseline demographics of patients (about overt HE)
8. Table S7. Univariate logistic regression analysis (about clinical factors)
9. Table S8. Univariate logistic regression analysis (about vascular factors)
10. Table S9. Identified factors for model construction
11. Table S10. Multivariate logistic regression analysis
12. Fig. S1. Model comparison
13. Fig. S2. Comparison among Model^C^, Model^V^, and Model^C-V^
14. Fig. S3. Subgroup analysis of Model^C-V^
15. Fig. S4. The AUCs and calibration of Model^C-V-ND^
16. Table S11. Pairwise comparison of the models
17. Table S12. Subgroup analysis of the AUCs
18. Table S13. Vascular factors of high-, middle-, and low-risk populations
19. Table S14. Clinical factors of high-, middle-, and low-risk populations
20. Table S15. Vascular morphologic changes in the with and without overt HE groups
21. Table S16. Shunt factors of patients in the training and validation datasets
22. **Table S1**

**Table S1. Clinical characterization of overt HE**

| Grade | Clinical characterization |
| --- | --- |
| II | Increased fatigue, apathy, flapping tremor/asterixis, ataxia, slurred speech |
| III | Somnolence, marked disorientation, rigor, stupor |
| IV | Coma |

HE: hepatic encephalopathy

1. **Table S2**

| **Table S2. Major parameters of CT image acquisition** | | | | | | | | | |
| --- | --- | --- | --- | --- | --- | --- | --- | --- | --- |
| **Hospitals** | **Scanner** | **TV (kV)** | **TC**  **(mA)** | **RT**  **(s)** | **DC**  **(mm)** | **FOV**  **(mm)** | **PM** | **Reconstruction** | **ST (mm)** |
| **NFH** | Philips Brilliance | 120 | 142 | 0.75 | 128×0.625 | 300×300 | 512×512 | Filter sharp (C) | 5 mm |
| **STCUAPH** | GE Optima | 120 | 280 | 0.80 | 64×0.625 | 400×400 | 512×512 | Filter Standard(B) | 5 mm |
| **ZSPH** | Philips Brilliance | 120 | 250 | 0.50 | 128×0.625 | 350×350 | 512×512 | Filter Standard(B) | 5 mm |
| **ZPH** | Siemens Somatom  Definition Flash | 120 | 160 | 0.50 | 64×0.625 | 350×350 | 512×512 | Filter sharp (C) | 2 mm  5 mm |
| **SPH** | Philips Brilliance | 120 | 250 | 0.50 | 64×0.625 | 500×500 | 1024×1024 | Filter sharp (C) | 5 mm |
| **SYSUTAH** | Toshiba Aquilion | 120 | 250 | 0.50 | 320x0.500 | 500×500 | 512×512 | Filter Standard(A) | 1 mm |
| TV: tube voltage; TC: tube current; RT: rotation time; DC: detector collimation; FOV: field of view; PM: pixel matrix; ST: slice thickness;  NFH: Nanfang Hospital; STCUAPH: The First Affiliated Hospital of the University of Science and Technology of China; ZSPH: Zhongshan City People's Hospital; ZPH: Zhuhai People’s Hospital SPH: Shenzhen People’s Hospital; SYSUTAH: The Third Affiliated Hospital of Sun Yat-sen University**.** | | | | | | | | | |

1. **Text S1: vascular measured methodology**

The diameters of the artery and portal venous system were measured in triplicate on the coronal arterial and portal venous phase images, and the average of three measurements was used as the diameter of the corresponding vessel. The measurement points of artery system diameters were the following: the internal diameter of the proper hepatic artery, splenic artery, and superior mesenteric artery were measured within 1 cm from its origin; the mean value was calculated and recorded as the internal diameter of the splenic artery.

The measurement points of the portal venous system diameters were the following: measured in the slice of the maximum diameter of the portal, splenic, and superior mesenteric veins; (2) the diameter of the right portal vein or left right portal vein were measured at the point where they bifurcated from the portal vein, respectively; and (3) the diameter of the left, middle, and right hepatic veins were measured at 1 cm away from the confluence of these three veins, respectively. In addition, the radiologists identified whether the paraumbilical vein, spleno-renal shunt, and gastric-renal shunt existed in each patient by reviewing the axial and coronal portal venous phase contrast-enhanced images working in consensus.

1. **Table S3**

| **Table S3.** **Evaluation of the intra- and interobserver agreements** | | |
| --- | --- | --- |
| **Vascular factors** | **Intra-observer ICC value** | **Inter-observer ICC value** |
| **Maximum diameter of the portal vein** | 0.929, (95% CI, 0.881-0.958) | 0.996, (95% CI, 0.993-0.998) |
| **Maximum diameter of the splenic vein** | 0.980 (95% CI, 0.967-0.988) | 0.980, (95% CI, 0.967-0.988) |
| **Maximum diameter of the superior mesenteric vein** | 0.943, (95% CI, 0.906-0.966) | 0.999, (95% CI, 0.999-1.000) |
| **Maximum diameter of the left portal vein** | 0.944, (95% CI, 0.904-0.967) | 0.944, (95% CI, 0.904-0.967) |
| **Maximum diameter of the right portal vein** | 0.975, (95% CI, 0.958-0.985) | 0.992, (95% CI, 0.986-0.995) |
| **Maximum diameter of the left hepatic vein** | 0.962, (95% CI, 0.880-0.983) | 0.996, (95% CI, 0.993-0.997) |
| **Maximum diameter of the middle hepatic vein** | 0.975, (95% CI, 0.946-0.987) | 0.995, (95% CI, 0.920-0.997) |
| **Maximum diameter of the right hepatic vein** | 0.973, (95% CI, 0.954-0.984) | 0.998, (95% CI, 0.997-0.999) |
| **Maximum diameter of the hepatic artery** | 0.943, (95% CI, 0.906-0.996) | 0.989, (95% CI, 0.982-0.994) |
| **Maximum diameter of the splenic artery** | 0.947, (95% CI, 0.874-0.974) | 0.929, (95% CI, 0.881-0/958) |
| **Maximum diameter of the superior mesenteric artery** | 0.950, (95% CI, 0.917-0.970) | 0.983, (95% CI, 0.971-0.990) |
| ICC: interclass correlation coefficient; 95% CI: 95% confidence interval. | | |

1. **Table S4**

| Table S4. Candidate clinical factors | |
| --- | --- |
| Classification | **Factor name** |
| Clinical factors |  |
|  | Age |
|  | Sex |
|  | Indication for TIPS |
|  | Diabetes |
|  | Cirrhosis etiology |
|  | Accompanying liver cancer |
|  | Hemoglobin |
|  | Platelet count |
|  | Aspartate aminotransferase |
|  | Alanine aminotransferase |
|  | Direct bilirubin |
|  | Indirect bilirubin |
|  | Serum sodium |
|  | Creatinine |
|  | Urea nitrogen |
|  | International normalized ratio |
|  | Activated partial thromboplastin time |
|  | Ammonia |
|  | Child–Pugh score |
|  | MELD score |

TIPS: transjugular intrahepatic portosystemic shunt

MELD: model for end-stage liver disease

1. **Table S5**

| **Table S5. Candidate vascular factors** | | |
| --- | --- | --- |
| **Classification of changes** | **Absolute value factor** | **ratio value factor** |
| **Diameter of vein** |  |  |
|  | Maximum diameter of the portal vein | Diameter ratio of the portal and splenic veins |
|  | Maximum diameter of the splenic vein | Diameter ratio of the splenic and superior mesenteric veins |
|  | Maximum diameter of the superior mesenteric vein | Diameter ratio of the portal and superior mesenteric veins |
|  | Maximum diameter of the left portal vein  Maximum diameter of the right portal vein | Diameter ratio of the left portal and right portal veins |
|  | Maximum diameter of the left hepatic vein | Diameter ratio of the left hepatic and portal veins |
|  | Maximum diameter of the middle hepatic vein | Diameter ratio of the middle hepatic and portal veins |
|  | Maximum diameter of the right hepatic vein | Diameter ratio of the right hepatic and portal veins |
| **Diameter of artery** |  |  |
|  | Maximum diameter of the hepatic artery | Diameter ratio of the splenic and hepatic arteries |
|  | Maximum diameter of the splenic artery |  |
|  | Maximum diameter of the superior mesenteric artery |  |
|  |  | Diameter ratio of the portal veins and hepatic artery |
|  |  | Diameter ratio of the splenic vein and artery |
|  |  | Diameter ratio of the superior mesenteric vein and artery |
| **Spontaneous shunting** |  |  |
|  | Spleno-renal shunt  Gastro-renal shunt  Paraumbilical vein shunt |  |
|  |  |  |
|  |  |  |
|  | | |

1. **Table S6**

| **Table S6. Baseline demographics of patients (about overt HE)** | | | |
| --- | --- | --- | --- |
| **Clinical factors** | **Without overt HE**  **(N=433)** | **With overt HE**  **(N=188)** | ***P*-value** |
| **Age** (year) | 52.0 ± 11.4 | 56.0 ± 11.0 | < 0.001* |
| **Sex** (N) |  |  | 0.938 |
| Male | 338 (78%) | 148 (79%) |  |
| Female | 95 (22%) | 40 (21%) |  |
| **Etiology** (N) |  |  | 0.859 |
| Alcohol | 149 (34%) | 59 (31%) |  |
| Hepatitis B/C | 207 (48%) | 95 (51%) |  |
| Cholestatic | 9 (2%) | 5 (3%) |  |
| Others | 68 (16%) | 29 (15%) |  |
| **Child–Pugh score** (point) | 8 (6, 9) | 8 (6, 9) | < 0.001* |
| **MELD score** (point) | 11 (8,13) | 11 (9,13) | < 0.001* |
| **ALT** (U/L) | 19.0 (14.0, 29.0) | 22.0 (15.0, 36.0) | 0.018* |
| **AST** (U/L) | 28.0 (21.0, 38.0) | 30.0 (23.0, 46.0) | 0.002* |
| **Direct bilirubin (**mg/dL**)** | 0.5 (0.3, 0.8) | 0.6 (0.4, 1.0) | 0.027* |
| **Indirect bilirubin (**mg/dL**)** | 0.5 (0.4, 0.8) | 0.5 (0.4, 0.8) | 0.948 |
| **Serum sodium** (mmol/L) | 140.0(138.0, 142.0) | 139.0 (136.0, 141.0) | < 0.001* |
| **INR** | 1.3 (1.2, 1.4) | 1.3 (1.2, 1.5) | 0.035* |
| **Ammonia** (μmol/L) |  |  | 0.143 |
| < 72.0 | 380 (88%) | 156 (83%) |  |
| ≥ 72.0 | 53 (12%) | 32 (17%) |  |
| **Indication for TIPS** (N) |  |  | 0.179 |
| Variceal bleeding | 362 (84%) | 148 (79%) |  |
| Refractory ascites | 71 (16%) | 40 (21%) |  |
| **Liver cancer** (N) |  |  | 0.032* |
| Yes | 51 (12%) | 35 (19%) |  |
| No | 382 (88%) | 153 (81%) |  |
| **Diabetes** (N) |  |  | 0.042* |
| Yes | 82 (19%) | 50 (27%) |  |
| No | 351 (81%) | 138 (73%) |  |
| * With a *P* < 0.050  Normally distributed factors are expressed using means ± standard deviations; non-normally distributed factors are expressed as medians (interquartile ranges).  ALT: Alanine aminotransferase; AST: Aspartate aminotransferase; INR: International normalized ratio; HE: hepatic encephalopathy; TIPS: transjugular intrahepatic portosystemic shunt. | | | |

1. **Table S7**

| Table S7. Univariate logistic regression analysis (about clinical factors) | | | |
| --- | --- | --- | --- |
| Classification | **Factor name** | **OR (95% CI)** | ***P*-value** |
| Clinical factors |  |  |  |
|  | Age | 0.974 (0.005-179.143) | 0.008* |
|  | Sex | 0.803 (0.142-4.551) | 0.376 |
|  | Indication for TIPS | 0.650 (0.028-15.344) | 0.107 |
|  | Diabetes | 0.716 (0.054-9.414) | 0.189 |
|  | Cirrhosis etiology | 1.024 (0.653-1.605) | 0.819 |
|  | Accompanying liver cancer | 0.636 (0.037-10.887) | 0.147 |
|  | Hemoglobin | 1.001 (0.611-1.640) | 0.801 |
|  | Platelet count | 1.004 (0.048-20.821) | 0.122 |
|  | Aspartate aminotransferase | 0.991 (0.005-217.372) | 0.006* |
|  | Alanine aminotransferase | 0.991 (0.014-69.832) | 0.030* |
|  | Direct bilirubin | 0.982 (0.018-52.257) | 0.043* |
|  | Indirect bilirubin | 1.003 (0.005-179.143) | 0.833 |
|  | Serum sodium | 1.107 (0.002-638.755) | 0.001* |
|  | Creatinine | 0.997 (0.081-12.319) | 0.200 |
|  | Urea nitrogen | 0.984 (0.314-3.078) | 0.561 |
|  | International normalized ratio | 0.255 (0.001-62.676) | 0.005* |
|  | Activated partial thromboplastin time | 0.969 (0.005-178.623) | 0.008* |
|  | Ammonia | 0.703 (0.052-9.559) | 0.183 |
|  | Child–Pugh score | 0.548 (0-2326399.928) | < 0.001* |
|  | MELD score | 0.932 (0.006-156.969) | 0.009* |

* With a *P* < 0.050

TIPS: transjugular intrahepatic portosystemic shunt; MELD: model for end-stage liver disease;

OR: odds ratio; CI: confidence interval.

1. **Table S8**

| Table S8. Univariate logistic regression analysis (about vascular factors) | | | | |
| --- | --- | --- | --- | --- |
| Classification | **Factor name** | | **OR (95% CI)** | ***P*-value** |
| Vascular factors | |  |  |  |
|  | Maximum diameter of the portal vein | | 0.876 (0.001-715.081) | 0.001* |
|  | Maximum diameter of the splenic vein | | 1.124 (0.001-943.069) | 0.001* |
|  | Maximum diameter of the superior mesenteric vein | | 1.003 (0.411-2.445) | 0.649 |
|  | Maximum diameter of the left portal vein | | 1.036 (0.207-5.198) | 0.411 |
|  | Maximum diameter of the right portal vein | | 1.049 (0.106-10.420) | 0.242 |
|  | Maximum diameter of the left hepatic vein | | 1.031 (0.324-3.285) | 0.554 |
|  | Maximum diameter of the middle hepatic vein | | 1.114 (0.019-66.897) | 0.037* |
|  | Maximum diameter of the right hepatic vein | | 1.093 (0.045-26.664) | 0.103 |
|  | Maximum diameter of the hepatic artery | | 0.902 (0.079-10.279) | 0.214 |
|  | Maximum diameter of the splenic artery | | 1.044 (0.347-3.142) | 0.574 |
|  | Maximum diameter of the superior mesenteric artery | | 0.877 (0.060-12.925) | 0.170 |
|  | Diameter ratio of the portal and splenic veins | | 0.189 (0-1128.024) | < 0.001* |
|  | Diameter ratio of the splenic and superior mesenteric veins | | 5.459 (0.002-11964.532) | < 0.001* |
|  | Diameter ratio of the portal and superior mesenteric veins | | 0.323 (0.004-25.806) | 0.025* |
|  | Diameter ratio of the left portal and right portal veins | | 0.609 (0.036-10.356) | 0.148 |
|  | Diameter ratio of the portal and left hepatic veins | | 4.110 (0.108-156.056) | 0.064 |
|  | Diameter ratio of the portal and middle hepatic veins | | 9.440 (0.023-3930.182) | 0.002* |
|  | Diameter ratio of the portal and right hepatic veins | | 7.553 (0.041-1396.442) | 0.008* |
|  | Diameter ratio of the hepatic and splenic arteries | | 1.422 (0.053-38.126) | 0.093 |
|  | Diameter ratio of the portal veins and hepatic artery | | 0.972 (0.509-1.855) | 0.741 |
|  | Diameter ratio of the splenic vein and artery | | 2.426 (0.002-2760.969) | < 0.001* |
|  | Diameter ratio of the superior mesenteric vein and artery | | 1.023 (0.475-2.204) | 0.696 |
|  | Spleno-renal shunt | | 0.692 (0.074-6.479) | 0.254 |
|  | Gastro-renal shun | | 0.720 (0.096-5.377) | 0.305 |
|  | Paraumbilical vein shunt | | 0.932 (0.496-1.751) | 0.748 |

* With a *P* < 0.050, OR: odds ratio; CI: confidence interval

1. **Table S9**

**Table S9. Identified factors for model construction**

| **Classification** | **Factor** |
| --- | --- |
| **Clinical model (Model^C^)** |  |
|  | Age |
|  | Child–Pugh score |
| **Vascular model (Model^V^)** |  |
|  | Maximum diameter of the portal vein |
|  | Diameter ratio of the portal and splenic veins |
|  | Diameter ratio of the portal and middle hepatic veins |
| **Combined model (Model^C-V^)** |  |
|  | Age |
|  | Child–Pugh score |
|  | Maximum diameter of the portal vein |
|  | Diameter ratio of the portal and splenic veins |

1. **Table S10**

**Table S10. Multivariate logistic regression analysis**

| **Classification** | **Factor** | **OR (95% CI)** | ***P*-value** | |
| --- | --- | --- | --- | --- |
| **Model^C^** |  |  | |  |
|  | Age | 1.014 (0.993-1.036) | | 0.182 |
|  | Child–Pugh score | 1.803 (1.547-2.100) | | < 0.001 |
| **Model^V^** |  |  | |  |
|  | Maximum diameter of the portal vein | 1.097 (1.011-1.191) | | 0.027 |
|  | Diameter ratio of the portal and splenic veins | 5.205 (2.878-9.415) | | < 0.001 |
|  | Diameter ratio of the portal and middle hepatic veins | 0.144 (0.030-0.699) | | 0.016 |
| **Model^C-V^** |  |  | |  |
|  | Age | 1.015 (0.993-1.038) | | 0.185 |
|  | Child–Pugh score | 1.825 (1.548-2.152) | | < 0.001 |
|  | Maximum diameter of the portal vein | 1.200 (1.099-1.310) | | < 0.001 |
|  | Diameter ratio of the portal and splenic veins | 3.500 (1.891-6.481) | | < 0.001 |

OR: odds ratio; CI: confidence interval

1. **Fig. S1. Model comparisons**


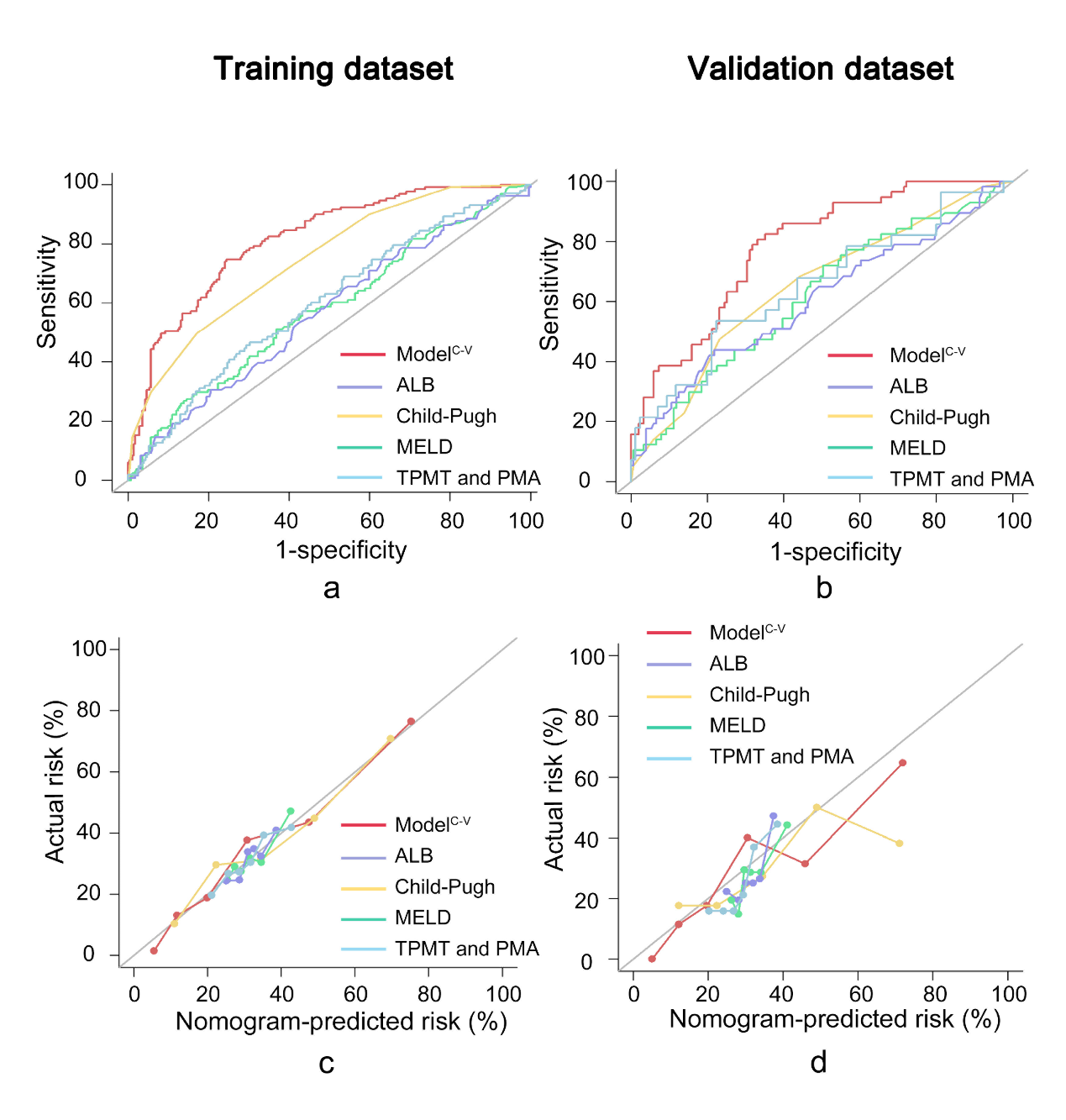


**Fig. S1. Model comparisons**

Comparison of Model^C-V^ with previous models. In terms of discrimination, the ROC curves (a, b) demonstrated significant improvement of Model^C-V^ both in the training dataset and validation dataset. Model^C-V^ also outperformed the other models on the calibration curves (c, d).

ALB, albumin; MELD, model for end-stage liver disease; TPMT, transversal psoas muscle thickness;

PMA, psoas muscle attenuation.

1. **Fig. S2. Comparison among Model^C^, Model^V^, and Model^C-V^**


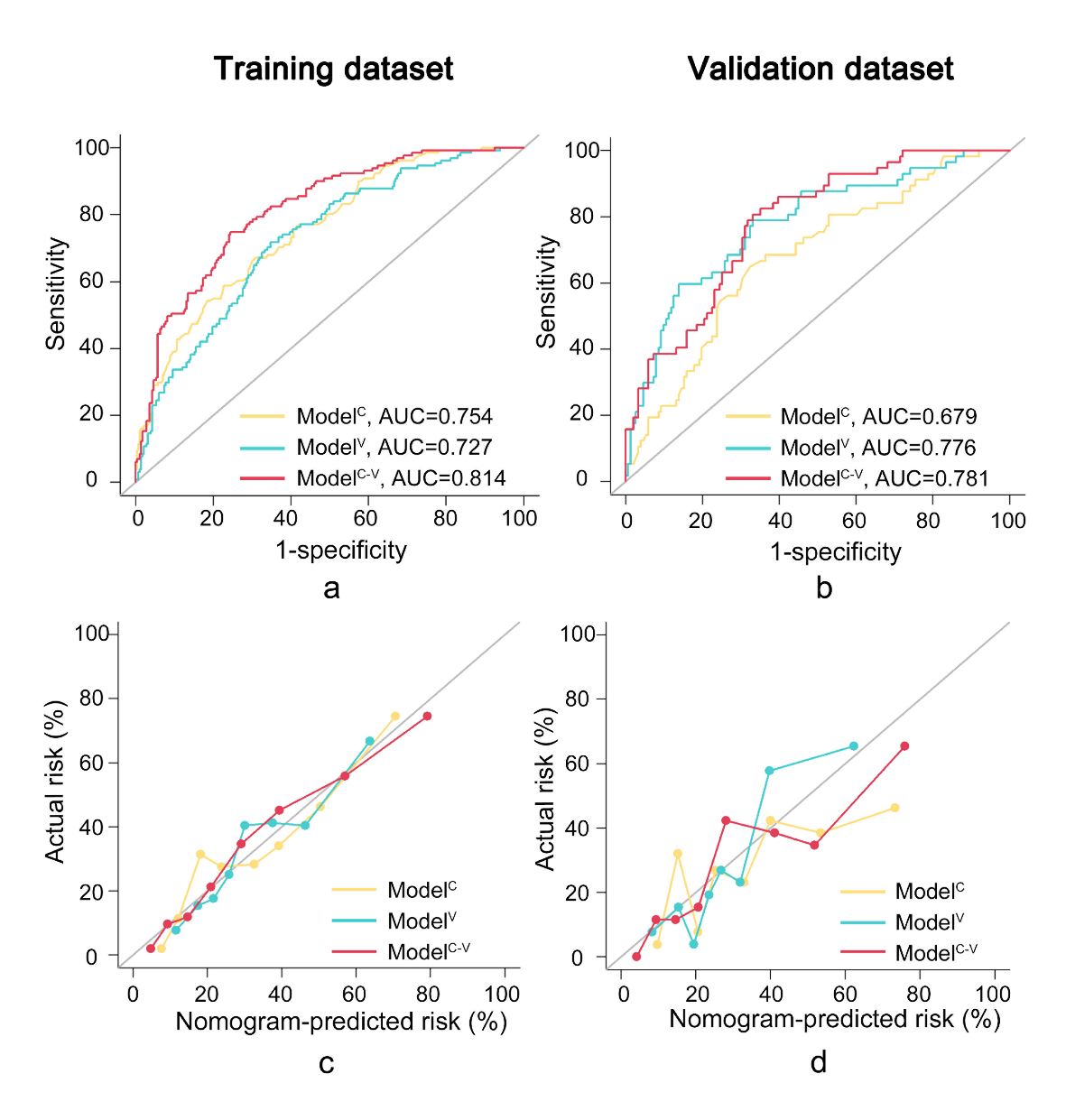


**Fig. S2. Comparison among Model^C^, Model^V^, and Model^C-V^**

To predict post-TIPS overt HE, the AUCs of the clinical, hepatic-associated vascular changes imaging, and combined models are shown for (a) the training dataset and (b) the validation dataset. The calibration of (c) the training dataset and (d) the validation dataset are shown.

TIPS: transjugular intrahepatic portosystemic shunt; HE: hepatic encephalopathy; AUC: area under the curve.

1. **Fig. S3. Subgroup analysis of Model^C-V^**


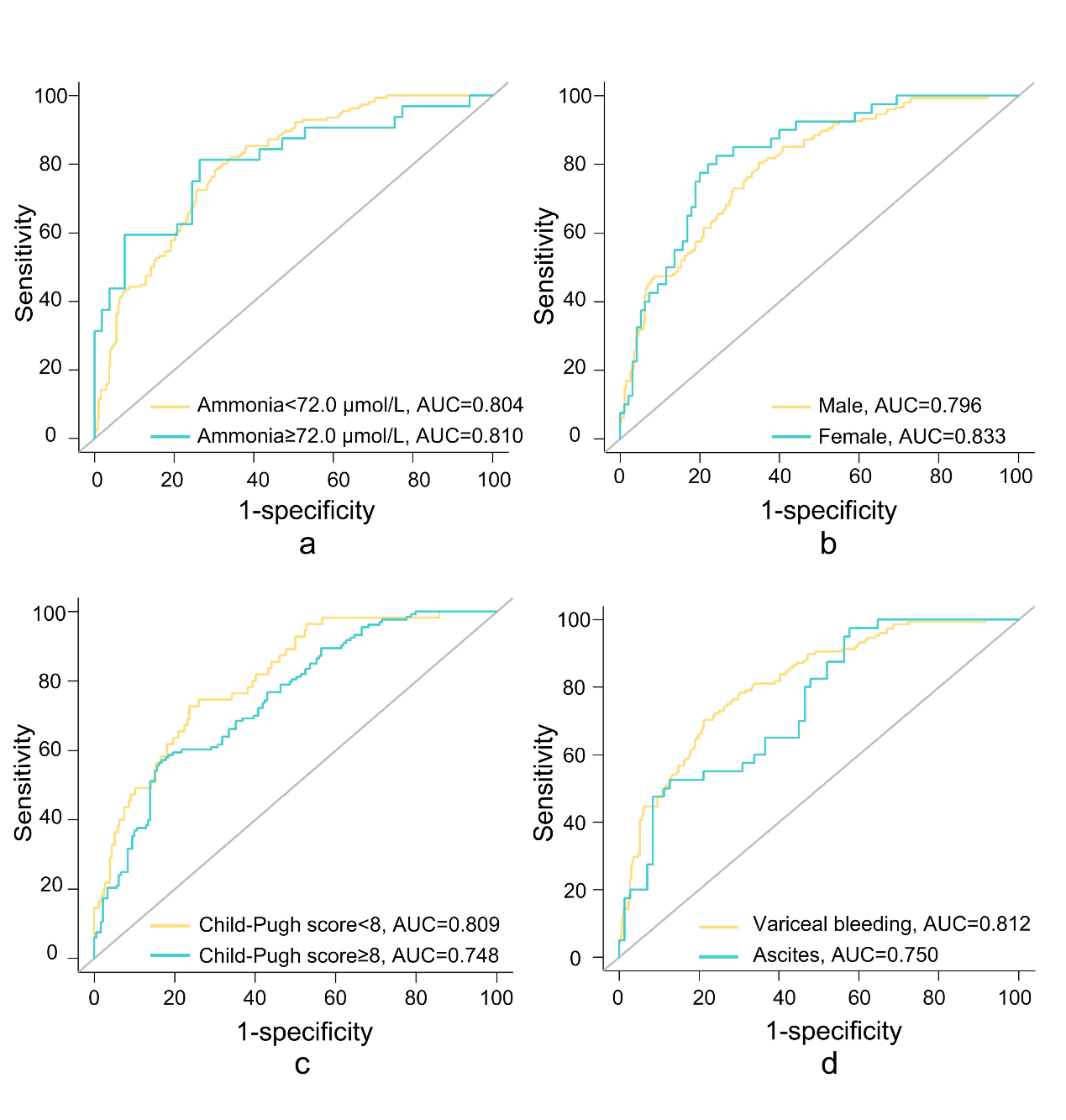


**Fig. S3. Subgroup analysis of Model^C-V^**

Subgroup analysis of Model^C-V^. The performance of the Model^C-V^ was not influenced by the (a) ammonia level, (b) sex, (c) Child–Pugh score, (d) and the indication for TIPS. TIPS: transjugular intrahepatic portosystemic shunt.

1. **Fig. S4 The AUCs and calibration of Model^C-V-ND^**


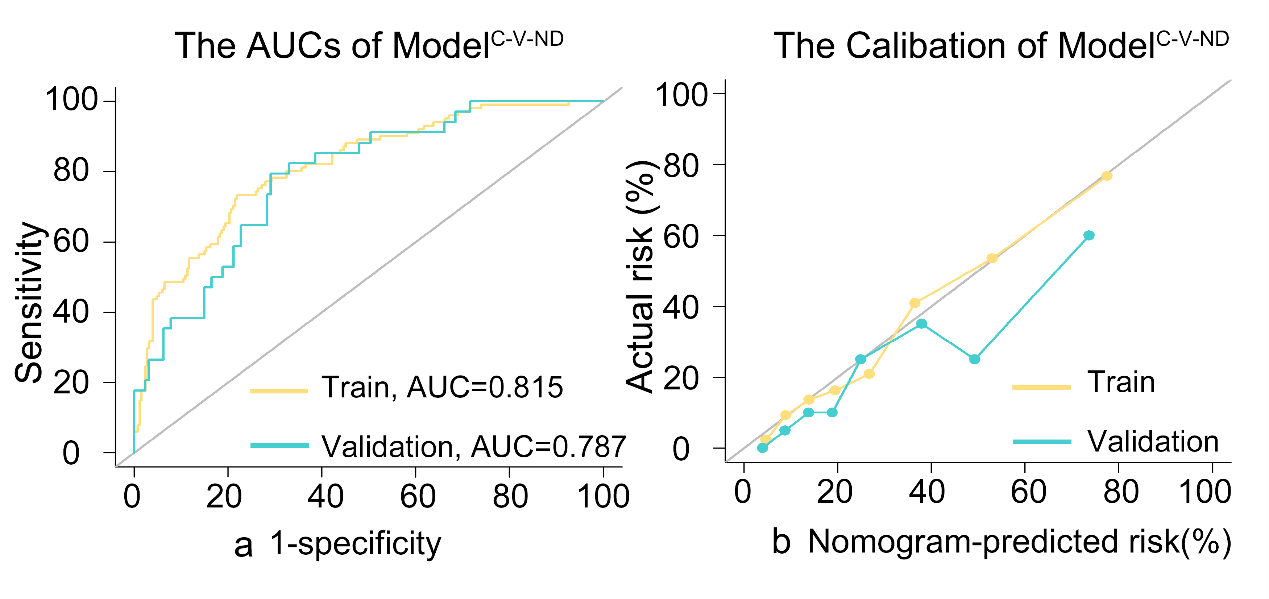


**Fig. S4 The AUCs and calibration of Model^C-V-ND^**

(a) The AUCs of Model^C-V-ND^ (the training dataset: 0.815 and the validation dataset: 0.787). (b) The calibration of Model^C-V-ND^.

1. **Table S11**

| **Table S11. Pairwise comparison of the models** | | | |
| --- | --- | --- | --- |
|  | **Delong test** | **NRI^1^** | **IDI^2^** |
| **Training dataset** |  |  |  |
| Model^C^ vs. Model^V^ | 0.448 | 0.166 | 0.034* |
| Model^C^ vs. Model^C-V^ | < 0.001* | < 0.001* | < 0.001* |
| Model^V^ vs. Model^C-V^ | < 0.001* | < 0.001* | < 0.001* |
| **Validation dataset** |  |  |  |
| Model^C^ vs. Model^V^ | 0.094 | 0.161 | 0.352 |
| Model^C^ vs. Model^C-V^ | < 0.001* | < 0.001* | < 0.001* |
| Model^V^ vs. Model^C-V^ | 0.905 | 0.177 | 0.028* |
| Data are presented as *P* values. * (*P* < 0.050 is significant)  ^1^NRI: net reclassification improvement; ^2^IDI: integrated discrimination improvement. | | | |

1. **Table S12**

| **Table S12. Subgroup analysis of the AUCs** | | |
| --- | --- | --- |
| **Subgroups divided by** | **Z** | ***P* value** |
| **Ammonia** (< 72.0 μmol/L vs. ≥ 72.0 μmol/L) | 0.110 | 0.913 |
| **Sex** (male vs. female) | 0.894 | 0.372 |
| **Child–Pugh score (**< 8 vs. ≥ 8**)** | 1.486 | 0.138 |
| **Indication for TIPS (**variceal bleeding vs. ascites**)** | 1.244 | 0.223 |

TIPS: transjugular intrahepatic portosystemic shunt; AUC: area under the curve

1. **Table S13**

| **Table S13. Vascular demographics of high-, middle-, and low-risk populations** | | | | |
| --- | --- | --- | --- | --- |
| **Vascular factors** | **Low risk**  **(N=209)** | **Middle risk**  **(N=201)** | **High risk**  **(N=211)** | ***P*-value** |
| **Maximum diameter of the portal vein** | 13.3 ± 2.6 | 14.6 ± 2.3 | 15.5 ± 3.0 | < 0.001* |
| **Maximum diameter of the splenic vein** | 12.1 ± 3.4 | 11.7 ± 3.2 | 10.2 ± 3.6 | < 0.001* |
| **Maximum diameter of the superior mesenteric vein** | 16.3 ± 7.8 | 12.2 ± 2.1 | 12.6 ± 2.2 | 0.534 |
| **Maximum diameter of the left portal vein** | 10.3 ± 2.6 | 10.8 ± 2.7 | 10.1 ± 2.7 | 0.044* |
| **Maximum diameter of the right portal vein** | 9.7 ± 2.6 | 10.0 ± 3.0 | 9.7 ± 3.0 | 0.506 |
| **Maximum diameter of the left hepatic vein** | 5.4 ± 2.0 | 5.5 ± 2.2 | 5.1 ± 2.3 | 0.226 |
| **Maximum diameter of the middle hepatic vein** | 5.2 ± 1.9 | 5.1 ± 2.0 | 5.0 ± 2.3 | 0.626 |
| **Maximum diameter of the right hepatic vein** | 4.9 ± 2.4 | 4.4 ± 1.8 | 4.2 ± 2.0 | 0.003* |
| **Maximum diameter of the hepatic artery** | 4.4 ± 1.3 | 4.4 ± 1.1 | 4.8 ± 1.4 | 0.214 |
| **Maximum diameter of the splenic artery** | 6.4 ± 1.3 | 6.3 ± 1.3 | 6.2 ± 1.5 | 0.422 |
| **Maximum diameter of the superior mesenteric artery** | 6.7 ± 1.0 | 7.1 ± 1.2 | 7.2 ± 1.2 | < 0.001* |
| **Spleno-renal shunt** |  |  |  | 0.122 |
| Yes | 32(15%) | 18(9%) | 23(11%) |  |
| No | 177(85%) | 183(91%) | 188(89%) |  |
| **Gastro-renal shun** |  |  |  | 0.532 |
| Yes | 27(13%) | 19(9%) | 23(11%) |  |
| No | 182(87%) | 182(91%) | 188(89%) |  |
| **Paraumbilical vein shunt** |  |  |  | 0.735 |
| Yes | 69(33%) | 68(34%) | 77(36%) |  |
| No | 140(67%) | 133(66%) | 134(64%) |  |
| * With a *P* < 0.050  Normally distributed factors are expressed using means ± standard deviations. | | | | |

1. **Table S14**

| **Table S14. Clinical demographics of high-, middle-, and low-risk populations** | | | | |
| --- | --- | --- | --- | --- |
| **Clinical factors** | **Low risk**  **(N=209)** | **Middle risk**  **(N=201)** | **High risk**  **(N=211)** | ***P*-value** |
| **Age** (year) | 49.0 ± 11.6 | 53.0 ± 10.8 | 55.5 ± 10.8 | < 0.001* |
| **Sex** (N) |  |  |  | 0.504 |
| Male | 158 (76%) | 159(79%) | 169(80%) |  |
| Female | 51 (24%) | 42(21%) | 42(20%) |  |
| **Etiology** (N) |  |  |  | 0.202 |
| Alcohol | 88 (42%) | 101 (50%) | 113 (54%) |  |
| Hepatitis B/C | 73 (35%) | 68 (34%) | 67 (32%) |  |
| Cholestatic | 5 (2%) | 4 (2%) | 5 (2%) |  |
| Others | 43 (21 %) | 28 (14%) | 67 (32%) |  |
| **Child–Pugh score** (point) | 6 (5, 7) | 8 (7, 8) | 9 (8, 10) | <0.001* |
| **MELD score** (point) | 9 (8,11) | 10 (9,12) | 12(10,14) | <0.001* |
| **ALT** | 20.0 (14.0, 29.0) | 20.0 (15.0, 31.0) | 21.0 (15.0, 32.0) | 0.300 |
| **AST** | 25.0 (20.0, 34.0) | 29.0 (21.0, 41.0) | 31.0 (22.0, 44.0) | 0.001* |
| **Direct bilirubin (**mg/dL**)** | 0.4 (0.3, 0.6) | 0.5 (0.3, 0.8) | 0.7 (0.4, 1.1) | <0.001* |
| **Indirect bilirubin (**mg/dL**)** | 0.5 (0.4, 0.8) | 0.5 (0.4, 0.8) | 0.6 (0.4, 1.0) | <0.001* |
| **Serum sodium** (mmol/L) | 140.0 (137.0, 142.0) | 140.0 (138.0, 142.0) | 139.0 (136.0, 141.0) | 0.115 |
| **INR** | 1.2 (1. 1, 1.3) | 1.3 (1.2, 1.4) | 1.4 (1.2, 1.6) | < 0.001* |
| **Ammonia** (μmol/L) |  |  |  | 0.005 |
| <72.0 | 186 (89%) | 181 (90%) | 169 (80%) |  |
| ≥72.0 | 23 (11%) | 27 (13%) | 29 (20%) |  |
| **Indication for TIPS**(N) |  |  |  | < 0.001* |
| Variceal bleeding | 191 (91%) | 175 (87%) | 144 (68%) |  |
| Refractory ascites | 18 (9%) | 26 (13%) | 67 (32%) |  |
| **Liver cancer** |  |  |  | 0.963 |
| Yes | 30 (14%) | 27 (13%) | 29 (14%) |  |
| No | 170 (86%) | 174 (87%) | 182 (86%) |  |
| **Diabetes** |  |  |  | < 0.001* |
| Yes | 26 (12%) | 44 (22%) | 62 (30%) |  |
| No | 183 (88%) | 157 (78%) | 149 (70%) |  |
| * With a *P* < 0.050  Normally distributed factors are expressed using means ± standard deviations; non-normally distributed factors are expressed as medians (interquartile ranges)  MELD: model for end-stage liver disease; ALT: Alanine aminotransferase; AST: Aspartate aminotransferase; INR: International normalized ratio; TIPS: transjugular intrahepatic portosystemic shunt. | | | | |

1. **Table S15**

| **Table S15. Vascular morphologic changes in the with and without overt HE groups** | | | | |
| --- | --- | --- | --- | --- |
| **Classification of changes** |  | **Without overt HE** | **With overt HE** | ***P*-value** |
| **Absolute value factor** |  |  |  |  |
|  | Maximum diameter of the portal vein | 14.1 ± 2.7 | 15.2 ± 2.8 | < 0.001* |
|  | Maximum diameter of the splenic vein | 11.8 ± 3.5 | 9.6 ± 3.1 | < 0.001* |
|  | Maximum diameter of the superior mesenteric vein | 14.3 ± 2.9 | 12.4 ± 2.3 | 0.605 |
|  | Maximum diameter of the left portal vein | 10.5 ± 2.6 | 10.2 ± 2.7 | 0.186 |
|  | Maximum diameter of the right portal vein | 9.9 ± 2.8 | 9.6 ± 3.1 | 0.187 |
|  | Maximum diameter of the left hepatic vein | 5.3 ± 2.2 | 5.1 ± 2.0 | 0.164 |
|  | Maximum diameter of the middle hepatic vein | 5.2 ± 2.1 | 4.9 ± 2.1 | 0.106 |
|  | Maximum diameter of the right hepatic vein | 4.6 ± 2.2 | 4.3 ± 1.9 | 0.066 |
|  | Maximum diameter of the hepatic artery | 4.5 ± 1.3 | 4.6 ± 1.2 | 0.535 |
|  | Maximum diameter of the splenic artery | 6.4 ± 1.4 | 6.2 ± 1.4 | 0.123 |
|  | Maximum diameter of the superior mesenteric artery | 6.9 ± 1.1 | 7.1 ± 1.2 | 0.059 |
| **ratio value factor** |  |  |  |  |
|  | Diameter ratio of the portal and splenic veins | 1.27 ± 0.36 | 1.59 ± 0.42 | < 0.001* |
|  | Diameter ratio of the splenic and superior mesenteric veins | 1.00 ±0 .32 | 0.85 ± 0.25 | < 0.001* |
|  | Diameter ratio of the portal and superior mesenteric veins | 1.20 ± 0.22 | 1.24 ± 0.22 | 0.039* |
|  | Diameter ratio of the left portal and right portal veins | 1.13 ± 0.58 | 1.15 ± 0.53 | 0.693 |
|  | Diameter ratio of the left hepatic and portal veins | 0.39 ± 0.17 | 0.34 ± 0.15 | 0.002* |
|  | Diameter ratio of the middle hepatic and portal veins | 0.38 ± 0.17 | 0.33 ± 0.16 | 0.001* |
|  | Diameter ratio of the right hepatic and portal veins | 0.34 ± 0.18 | 0.29 ± 0.14 | 0.001* |
|  | Diameter ratio of the splenic and hepatic arteries | 1.52 ± 0.54 | 1.43 ± 0.45 | 0.037* |
|  | Diameter ratio of the portal veins and hepatic artery | 3.45 ± 1.23 | 3.46 ± 1.16 | 0.927 |
|  | Diameter ratio of the splenic vein and artery | 1.89 ± 0.56 | 1.67 ± 0.43 | < 0.001* |
|  | Diameter ratio of the superior mesenteric vein and artery | 2.13 ± 0.95 | 1.78 ± 0.40 | 0.552 |

* With a *P* < 0.050

HE: hepatic encephalopathy.

1. **Table S16**

| **Table S16.** **Shunt factors of patients in the training and validation datasets** | | | |
| --- | --- | --- | --- |
| **Factors** | **Training dataset**  **(N=413)** | **Validation dataset**  **(N=208)** | ***P*-value** |
| **Spleno-renal shunt** (N) |  |  | 0.254 |
| Yes | 46 (11%) | 27 (13%) |  |
| No | 367 (89%) | 181 (87%) |  |
| **Gastro-renal shun** (N) |  |  | 0.305 |
| Yes | 47 (11%) | 22 (11%) |  |
| No | 366 (89%) | 186 (89%) |  |
| **Paraumbilical vein shunt** (N) |  |  | 0.748 |
| Yes | 153 (37%) | 61 (29%) |  |
| No | 260 (67%) | 147 (71%) |  |
| * With a *P* < 0.050. | | | |
